# New Insights Into the Antibacterial Mechanism of Cryptotanshinone, a Representative Diterpenoid Quinone From *Salvia miltiorrhiza* Bunge

**DOI:** 10.3389/fmicb.2021.647289

**Published:** 2021-02-25

**Authors:** Bo-Chen Chen, Zhi-Shan Ding, Jian-Sheng Dai, Ni-Pi Chen, Xing-Wen Gong, Lie-Feng Ma, Chao-Dong Qian

**Affiliations:** ^1^College of Life Science, Institute of Molecular Medicine, Zhejiang Chinese Medical University, Hangzhou, China; ^2^College of Medical Technology, Zhejiang Chinese Medical University, Hangzhou, China; ^3^Department of Biological Engineering, Zhejiang Gongshang University, Hangzhou, China; ^4^College of Pharmaceutical Science, Zhejiang University of Technology, Hangzhou, China

**Keywords:** cryptotanshinone, respiratory chain inhibitor, menaquinone, type II NADH:quinone dehydrogenase, metabolome analysis

## Abstract

The rapid rise of antibiotic resistance causes an urgent need for new antimicrobial agents with unique and different mechanisms of action. The respiratory chain is one such target involved in the redox balance and energy metabolism. As a natural quinone compound isolated from the root of *Salvia miltiorrhiza* Bunge, cryptotanshinone (CT) has been previously demonstrated against a wide range of Gram-positive bacteria including multidrug-resistant pathogens. Although superoxide radicals induced by CT are proposed to play an important role in the antibacterial effect of this agent, its mechanism of action is still unclear. In this study, we have shown that CT is a bacteriostatic agent rather than a bactericidal agent. Metabolome analysis suggested that CT might act as an antibacterial agent targeting the cell membrane. CT did not cause severe damage to the bacterial membrane but rapidly dissipated membrane potential, implying that this compound could be a respiratory chain inhibitor. Oxygen consumption analysis in staphylococcal membrane vesicles implied that CT acted as respiratory chain inhibitor probably by targeting type II NADH:quinone dehydrogenase (NDH-2). Molecular docking study suggested that the compound would competitively inhibit the binding of quinone to NDH-2. Consistent with the hypothesis, the antimicrobial activity of CT was blocked by menaquinone, and the combination of CT with thioridazine but not 2-n-heptyl-4-hydroxyquinoline-*N*-oxide exerted synergistic activity against *Staphylococcus aureus*. Additionally, combinations of CT with other inhibitors targeting different components of the bacterial respiratory chain exhibit potent synergistic activities against *S. aureus*, suggesting a promising role in combination therapies.

## Introduction

Infectious diseases remain a major threat to global health due to widespread antibiotic resistance among pathogens. Although improved preventive measures have reduced resistance in some bacteria, the discovery and development of new antimicrobial agents with a unique mechanism of action has been and will continue to be necessary for the treatment of infections caused by novel drug-resistant pathogens. For a long time, the clinically used antibiotics mainly affect five major targets or biosynthetic pathways: the biosynthesis of DNA, RNA, proteins, peptidoglycan, and folic acid (Hurdle et al., 2011). Recently, the respiratory system has emerged as an attractive target for the development of new antibiotics, especially against multidrug-resistant tuberculosis (Hards and Cook, 2018; Iqbal et al., 2018). The mitochondrial respiratory systems of mammal usually consist of type I NADH dehydrogenase (NDH-1), succinate dehydrogenase, cytochrome bc_1_ complex, cytochrome c oxidase, and ATP synthase. However, for adaption to different environments, the respiratory system of bacteria is far more diverse than that of mitochondria (Anraku, 1988). For instance, *Mycobacterium tuberculosis* can encode two distinct terminal oxidases as well as two types of NADH dehydrogenases, NDH-1 and type II NADH dehydrogenase (NDH-2) (Hards and Cook, 2018), while *Bacillus subtilis* possesses one NDH-2 and four types of terminal oxidases (Azarkina et al., 1999; Melo et al., 2004).

Numerous antimicrobial molecules targeting different components of the respiratory chain have been discovered. Thioridazine targets NDH-2 to block the electron transfer chain of bacteria and is effective against *Staphylococcus aureus* and *M. tuberculosis* (Weinstein et al., 2005; Schurig-Briccio et al., 2014). Bedaquiline and its analog TBAJ-876 kill *M. tuberculosis* by specifically inhibiting F_1_F_0_-ATP synthase (Andries, 2005; Koul et al., 2007; Sarathy et al., 2019). Lysocin E exerts its antimicrobial activity through a direct interaction with menaquinone (MK), which is the sole quinone in most of the Gram-positive bacteria, including *M. tuberculosis*, *S. aureus*, and *B. subtilis* (Hamamoto et al., 2015; Paudel et al., 2016; Boersch et al., 2018). Moreover, many compounds that target MK biosynthesis (Paudel et al., 2016; Boersch et al., 2018) and QcrB, a component of the cytochrome bc_1_ complex (Foo et al., 2018; Iqbal et al., 2018; Lu et al., 2019), have been described. It is worth mentioning that bedaquiline was approved by the FDA in 2012 for the treatment of pulmonary multidrug-resistant tuberculosis in adults, and Q203, an imidazopyridine amide compound targeting the respiratory cytochrome bc_1_ complex of *M. tuberculosis*, has recently entered clinical trials (Iqbal et al., 2018).

Over the past few decades, much attention has been directed to the study of plant-derived compounds due to their antibacterial activities, especially against multidrug-resistant pathogenic bacteria (Cha et al., 2014; Barbieri et al., 2017; Teng et al., 2018). Cryptotanshinone (CT), a representative diterpenoid quinone isolated from the root of *Salvia miltiorrhiza* Bunge (Dan Shen), is one of such compounds. It is the major active constituents of Danshentong capsules and Kecuoyintone gel, which are Chinese patent medicines used for the treatment of disorders such as acne vulgaris and other skin infections (Zhang et al., 2003; Zhao et al., 2007). CT exhibits antibacterial activity against a wide range of Gram-positive pathogenic bacteria (Lee et al., 1999; Lu, 2020). Clinically isolated methicillin-resistant *S. aureus* and vancomycin-resistant *S. aureus* were also reported to be sensitive to CT (Cha et al., 2014), making it an attractive new antibiotic candidate. However, little information has been obtained about the mode of action (MoA) of CT. It has only been speculated that superoxide radicals induced by CT might be important in the antibacterial activity of the agent (Lee et al., 1999; Feng et al., 2009).

To gain novel insight into the MoA of this compound, we conducted a comprehensive study using a model organism *B. subtilis* and an opportunistic pathogen *S. aureus*, the latter of which causes a wide range of hospital- and community-acquired infections (Al-Mebairik et al., 2016). We found that CT was a respiratory chain inhibitor probably by targeting NDH-2. Furthermore, we demonstrated that the phytochemical was strongly synergistic with several respiratory chain inhibitors.

## Materials and Methods

### Chemicals and Bacterial Strains

Cryptotanshinone (≥98%, HPLC), menaquinone-4 (MK4), and NADH were purchased from Sigma–Aldrich (St. Louis, MO, United States). Reagents used in metabolomics: methanol, formic acid, and acetonitrile were purchased from CNW Technologies GmbH (Germany). Unless otherwise stated, all agents were dissolved in DMSO and then diluted to ensure a final DMSO concentration of ≤3.2% (vol/vol). *S. aureus* ATCC 25923, *S. aureus* ATCC 29213, *S. aureus* ATCC 43300, and *B. subtilis* 168 (Chen et al., 2018) were used in this study.

### Antimicrobial Testing

The minimum inhibitory concentration (MIC)/minimum bactericidal concentration (MBC) was determined as described previously (Chen et al., 2018). Time-kill experiments were performed to evaluate the bactericidal/bacteriostasis activity of CT against *S. aureus* ATCC 43300 and *B. subtilis* 168 (Chen et al., 2018). Vancomycin was used as a positive control of bactericidal antibiotic, and DMSO (3.2%) was used as a negative control.

### Untargeted Metabolomics Analysis

Overnight culture of *B. subtilis* was inoculated in Mueller–Hinton (MH) broth and incubated to early logarithmic phase. Bacterial cells were then diluted to OD_600_ = 0.2 with fresh MH broth, and treated with 16 μg/mL CT or 1.6% DMSO for 2 h. The cells were harvested by centrifugation, washed, and resuspended with ice-cold saline. The suspended cells were divided, pelleted, and then frozen rapidly in liquid nitrogen for 5 min, followed by storage at −80°C until analysis. An accurately weighed sample was resuspended in 1mL methanol:water (4:1 = v:v), added to 20 μL of 2-chloro-l-phenylalanine (0.3 mg/mL) dissolved in methanol as internal standard, and transferred to a 2.0-mL glass vial. A total of 200 μL of chloroform was added to each vial. The cells were then broken by sonication on an ice bath for 6 min at 500 W and centrifuged (16,000 × *g*, 4 °C) for 10 min. Quality control samples were prepared by mixing aliquots of all samples that served as a pooled sample. One milliliter of the supernatant was transferred to a 1.5-mL Eppendorf tube and dried in a freeze-concentration centrifugal dryer. The sample was then resuspended in 400 μL methanol: water (7:3 = v:v). After centrifugation, 150 μL of the supernatant was filtered through a 0.22 μm filter, followed by LC-MS analysis. ACQUITY UPLC I-Class system coupled with VION IMS QTOF mass spectrometer (Waters Corporation, Milford, CT, United States) was used to analyze the metabolic profiling in both ESI positive and ESI negative ion modes. The acquired LC-MS raw data were analyzed by the progenesis QI software (Waters Corporation, Milford, CT, United States). Principal component analysis (PCA), partial least squares-discriminant analysis (PLS-DA), and orthogonal partial least-squares-discriminant analysis (OPLS-DA) were carried out to visualize the metabolic alterations among experimental groups, after mean centering and Pareto variance scaling, respectively. Variable importance in the projection (VIP) ranked the overall contribution of each variable to the OPLS-DA model, and those variables with VIP > 1 were considered relevant for group discrimination. Student’s *t*-test and fold change analyses were used to compare the difference of metabolites between the two groups.

### Analysis of CT-Induced Release of Cytoplasmic Components

The intracellular concentration of potassium/magnesium in bacteria was determined as previously reported (Müller et al., 2016). Element concentrations were determined by iCAP 6300 Spectrometer (Thermo Fisher Scientific, United States) and normalized to the DMSO control. For analysis of ATP release, cells of *B. subtilis* 168 (∼10^8^ CFU/mL) were resuspended in PBS and incubated with antibiotics at 37°C for 30 min. The samples were then centrifuged at 8,000 × *g* at 4°C for 10 min. A total of 100 μL of supernatant was mixed with an equal amount of working reagent from the BacTiter glow ATP detection Kit (Promega, United States) and incubated for 2 min. Luminescence was measured subsequently with a FLUOStar Omega (BMG LABTECH GmbH, Germany).

### Propidium Iodide Stain

The cells of bacteria were grown to logarithmic phase and washed twice with PBS. The cell suspensions were then incubated with CT at 37°C for 2 h before centrifuging at 6,000 × *g* for 10 min. The acquired cells were resuspended in PBS and incubated with 30 μM propidium iodide (PI) at 37°C for 30 min. Fluorescence intensity was measured by an Accuri C6 flow cytometry with excitation at 490 nm and emission at 635 nm.

### Determination of Membrane Potential

The effects of CT on bacterial membrane potential were determined first with a flow cytometer using fluorescent dye 3,3′-diethyloxacarbocyanine iodide [DiOC_2_(3)] (Molecular Probes, Fisher Scientific). Bacteria grown to logarithmic growth phase were harvested and resuspended in PBS, and treated with drug and DiOC_2_(3) (10 μM) for 10 min at room temperature. Stained bacteria were assayed in a CytoFlex S flow cytometer (Beckman Coulter, United States) with a laser emitting at 485 nm. Fluorescence was observed in the green and red channels according to the instructions of the BacLight^TM^ Bacterial Membrane Potential Kit (Thermo Fisher Scientific, United States).

In investigating whether the effect of CT on bacterial membrane potential was time-dependent, the resuspended cells of bacteria in PBS were added into 96-well plates, incubated with 10 μM DiOC_2_(3) for 10 min in the dark, and the baseline recorded for 2 min using a FLUOstar Omega. Fluorescence intensity was measured at an excitation wavelength of 485 nm and two emission wavelengths, 530 nm (green) and 630 nm (red) (Saising et al., 2018). Subsequently, drugs were added, and fluorescence was measured for a specified time at room temperature.

### Preparation of Inverted Membrane Vesicles

Membrane vesicles were isolated from the overnight cultures of *S. aureus* ATCC 43,300 (Lamontagne Boulet et al., 2018). The cells were suspended in KPN (20 mM potassium phosphate, 140 mM NaCl, pH 7.2), disrupted in a French press at 16,000 psi, and centrifuged at 6,000 × *g* for 30 min. The supernatant was centrifuged at 150,000 × *g* for 40 min using an ultracentrifuge (Optima XPN-100, Beckman, United States). The pellets were resuspended and centrifuged at 12,000 × *g* for 30 min. The supernatant containing membrane vesicles was stored at −80 °C. Protein concentration was estimated by a BCA protein assay kit (Beyotime Biotechnology, Shanghai, China) using bovine serum albumin as a standard.

### ATP Synthesis Assay

Bacteria (∼10^6^ CFU/mL) were preincubated with CT under stirring conditions for 10 min. Subsequently, 10 mM glucose (final concentration) was added, and the mixture further incubated for 30 min. Bacterial suspensions (100 μL) were then transferred into 96-well plates and mixed with an equal amount of working reagent from the BacTiter glow ATP detection Kit. Luminescence was finally measured with a FLUOStar Omega after incubation for 5 min. The effect of CT on ATP synthesis by membrane vesicles isolated from *S. aureus* was determined as previously described (Lamontagne Boulet et al., 2018).

### Oxygen Consumption Assay

Oxygen consumption by membrane vesicles of *S. aureus* was measured polarographically with a clark-type polarographic electrode (Strathkelvin 782 dissolved oxygen meter, United Kingdom) (Weinstein et al., 2005). Membrane vesicles (0.2 mg/mL) were suspended in KPN buffer at 37°C, and 1 mM NADH was added to initiate respiration. Subsequently, CT was added to the reaction mixture, followed by the addition of dithiothreitol and ubiquinone-10. The result was expressed as the relative oxygen content, and the initial oxygen concentration was set as 100%.

### Bacterial NADH/NAD^+^ Ratios Assay

NADH/NAD^+^ ratios were determined using an Amplite fluorimetric NAD/NADH ratio assay kit (AAT Bioquest, Inc., United States). Bacteria were grown to the early logarithmic phase and collected by centrifugation. The cell pellets were resuspended and treated with drug for 30 min at 37°C. The cells were pelleted at 6,000 × *g* for 5 min, washed, and resuspended with lysis buffer. Measurement of the NADH/NAD^+^ ratios in the supernatant was performed according to the manufacturer’s instructions.

### Molecular Docking Simulation

Molecular docking using Autodock 4.2 was employed to explore the mode of binding of CT with NDH-2. The X-ray crystal structure of NDH-2 from *S. aureus* (PDB ID: 4XDB) was retrieved from the Protein Data Bank^1^. All crystallographic water molecules and ions were removed from the protein structure. The 3-D structures of CT and ubiquinone-5 were retrieved from the PubChem database. The conformations were generated using the best conformational analysis method with CHARMM force field parameters. The obtained conformations were then docked into the binding site of NDH-2. The docked conformation with the lowest energy was used for the analysis of binding mode.

## Results

### CT Is a Bacteriostatic Agent

**FIGURE 1:**
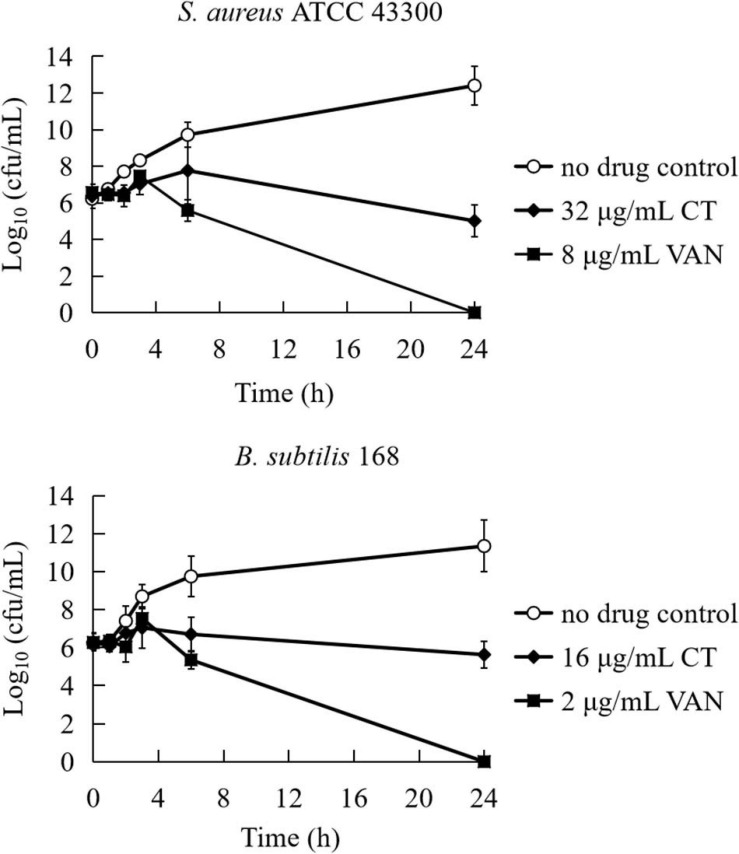
Time-kill curves for *Bacillus subtilis* and *Staphylococcus aureus* treated with CT. The curves are viable cell concentrations plotted against time. MIC, minimum inhibitory concentration; VAN, vancomycin. DMSO (3.2%) is as a no drug control. Data represent the mean ± SD (*n* = 3).

To evaluate the bactericidal/bacteriostatic behavior of the agent, the MIC and MBC were determined for four strains (Table 1). The MICs of CT against tested bacteria ranged from 4 to 16 μg/mL. The MBC of CT for each strain was >64 μg/mL, and the MBC/MIC ratios were all higher than 4, indicating that CT is a bacteriostatic agent rather than a bactericidal agent (Clinical and Laboratory Standards Institute, 2009). To further examine the bacteriostatic activities of CT, time-kill assays against *S. aureus* ATCC 43300 and *B. subtilis* 168 were performed. As shown in Figure 1, less than one logarithmic unit of killing was observed at 4 × MIC of CT against two stains for 24 h, which was consistent with the bacteriostatic behavior revealed by the MBC/MIC analysis described above.

**TABLE 1:** The MICs and MBCs of CT against reference strains.

**Strains and compounds**	**MIC (μg/mL)**	**MBC (μg/mL)**	**MBC/MIC**
***S. aureus* ATCC 29213**			
CT	16	>64	>4
Vancomycin	1	2	2
***S. aureus* ATCC 43300**			
CT	8	>64	>8
Vancomycin	2	2	1
***S. aureus* ATCC 25923**			
CT	8	>64	>8
Vancomycin	1	1	1
***B. subtilis* 168**			
CT	4	>64	>16
Vancomycin	0.5	>1	>2

### CT Induces Significant Changes in the Membrane Phospholipids

To obtain a first hint as to the antibacterial mechanism of CT, we conducted an untargeted metabolomics study. PCA and heatmaps showed that the pooled biological quality control samples were tightly clustered together, indicating minimal technical variation. Under two treatment conditions, a total of 4,444 putative metabolites were identified. The PCA (Supplementary Figure 1A) and PLS-DA (Supplementary Figure 1B) showed that the untreated control and CT-treated samples were significantly separated. OPLS-DA (Supplementary Figures 1C,D) and two-tailed Student’s *t*-test further revealed that 406 metabolites were significantly altered (VIP > 1, *p* < 0.05) following treatment with CT (Supplementary Data Set 1).

**FIGURE 2:**
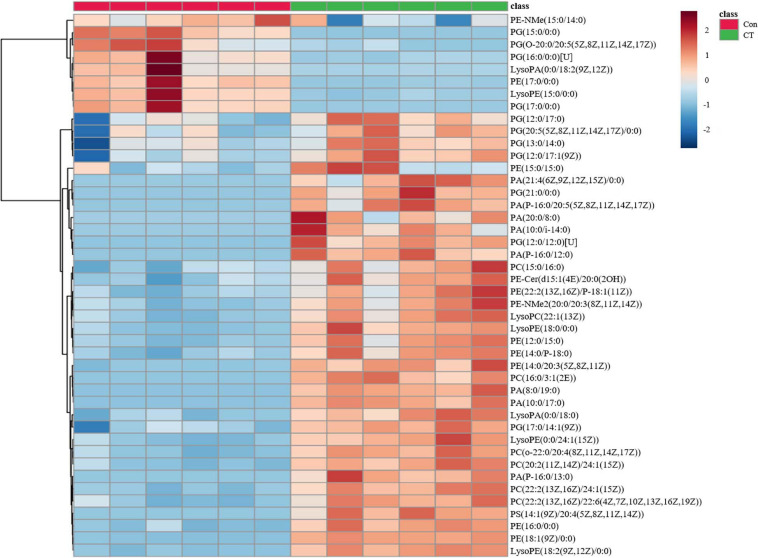
CT significantly affects the glycerophospholipid metabolism of *Bacillus subtilis*. DMSO is as a control without drug (Con). Deep red represents the highest level, and deep blue represents the lowest level, with white representing equal levels. Each group is identified by green (CT) and red (DMSO) bars at the top of each column. Six biological replicates were tested for each sample.

The most prominent changes associated with a specific metabolic pathway were mapped to KEGG pathways. Metabolites in purine metabolism, arginine biosynthesis, glycerophospholipid metabolism, sphingolipid metabolism, ABC transporters, biosynthesis of amino acids, and D-glutamine and D-glutamate metabolism were found to significantly differ between control and CT treatment (*p* < 0.01; Supplementary Figure 2). In general, CT dramatically decreased metabolites related to purine, pyrimidine, and amino acid metabolism. The relative abundance of nicotinamide adenine dinucleotide (NAD) was also significantly decreased in CT group (log_2_FC = −6.5; *p* < 0.01). Interestingly, CT induced significant changes in a wide range of the major membrane phospholipids in *B. subtilis*, including phosphatidylglycerol (PG), phosphatidylethanolamine (PE), and phospholipid acid (PA). More concretely, CT enriched a number of phospholipids {PE (15:0/15:0), PE (16:0/0:0), PE [18:1(9Z)/0:0], Lyso PE (15:0/0:0), PA (10:0/17:0), PA (8:0/19:0), PA (P-16:0/13:0), and PA (P-16:0/12:0)} but decreased the levels of PG (15:0/0:0), PG (16:0/0:0)[U], and PG (17:0/0:0) (Figure 2 and Supplementary Data Set 2). Many of metabolites affected by CT were related to glycerophospholipid, suggesting that CT might act as an antibacterial agent targeting the cell membrane.

### CT Does Not Cause Severe Bacterial Membrane Damage but Dissipates Membrane Potential

**FIGURE 3:**
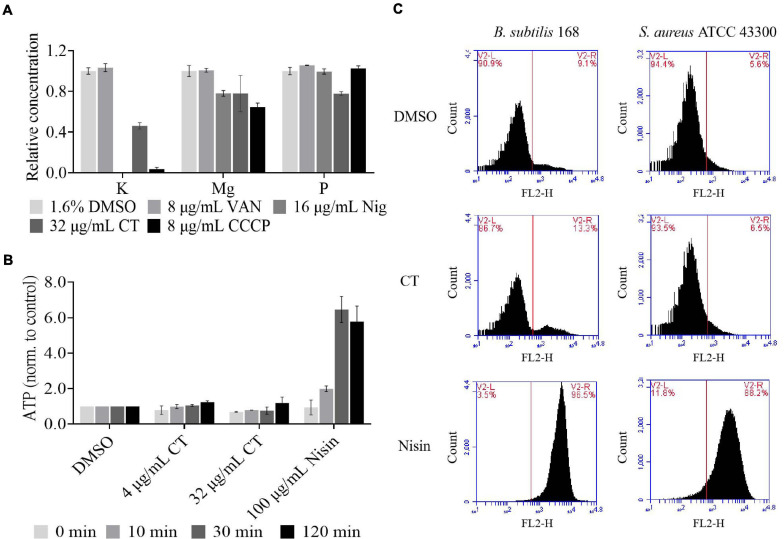
Effect of CT on bacterial membrane integrity. **(A)** Determination of cellular element concentrations of *B. subtilis* 168 by inductively coupled plasma optical emission spectroscopy after CT treatment for 30 min. DMSO, non-drug treated control; VAN (vancomycin), negative control; Nig (nigericin), specific potassium ionophore; CCCP, depolarization agent. The experiments were carried out in triplicate in two independent replications. **(B)** Leakage of ATP following exposure of *B. subtilis* 168 to antimicrobial agents. Nisin, a pore-forming agent. Data are presented as mean ± SD of three independent experiments with triplicate measurements. **(C)** Staining with propidium iodide. Representative data from three independent cultures of *S. aureus* ATCC 43300 and *B. subtilis* 168 were shown following exposure to antimicrobial agents at 8 × MIC for 120 min.

Antimicrobial agents targeting cell membranes usually include membrane structure disruptors and respiratory chain inhibitors (Hurdle et al., 2011). To distinguish the type of action of CT, the effects of this compound on bacterial membrane integrity were investigated. We first investigated whether it causes the release of cytoplasmic components. As shown in Figure 3A, partial leakage of potassium and magnesium was observed when *B. subtilis* was treated with CT for 30 min. However, CT did not cause the release of ATP even after 120 min treatment with 32 μg/mL (Figure 3B), implying that CT does not cause severe membrane disruption. To confirm this finding, *S. aureus* and *B. subtilis* were stained with a fluorescent dye PI, an indicator of severe membrane disruption. When two strains were treated with nisin, marked increases in fluorescence were observed compared to the untreated cultures (Figure 3C). In contrast, incubation of cultures with 8 × MIC of CT for 120 min did not result in the uptake of PI (Figure 3C), indicating that CT did not cause serious damage to the bacterial membrane.

**FIGURE 4:**
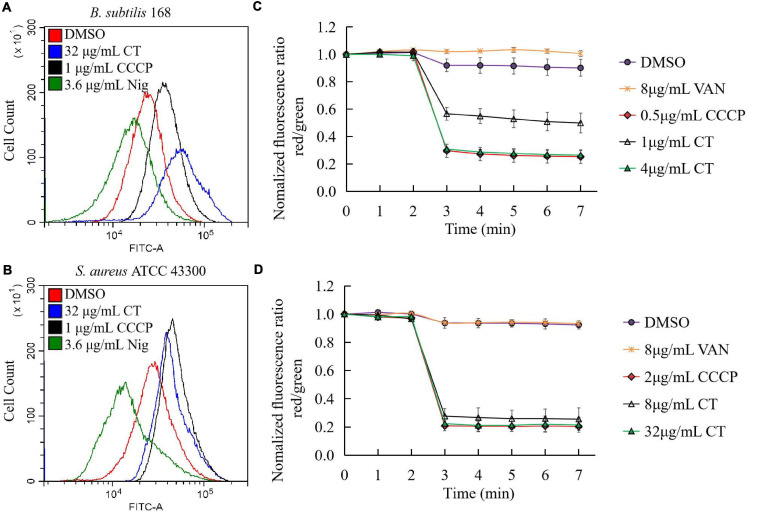
Effect of CT on membrane potential of *B. subtilis* 168 **(A,C)** and *S. aureus* ATCC 43300 **(B,D)**. **(A,B)** Membrane potential estimation by flow cytometry using DiOC_2_(3) dye; Representative data from three independent cultures of both strains were shown following exposure to different agents for 10 min. **(C,D)** Fluorescence of DiOC_2_(3) was detected at an excitation wavelength of 485 nm and two emission wavelengths, 530 nm (green) and 630 nm (red), using a microplate reader. Addition of CT to the cell suspensions of bacteria is indicated by an arrow. Data are presented as mean ± SD of three independent experiments. DMSO (1.6%), non-drug treated control; CCCP, depolarization control; Nigericin, hyperpolarization control; VAN (vancomycin), negative control.

We then investigated the effect of CT on bacterial membrane potential. First, we evaluated the ability of CT to induce membrane depolarization using DiOC_2_(3). The green, fluorescent dye formed red fluorescent aggregates with increasing membrane potential. When the membrane potential is disrupted with a small molecule, the dye is released into the medium resulting in an increase in green fiuorescence (Novo et al., 2000). As expected, the addition of CCCP to the suspensions of *B. subtilis* or *S. aureus* caused a marked increase in green fluorescence, while incubation of both strains with nigericin decreased the green fluorescence intensity (Figures 4A,B). Similar to CCCP, CT dissipated the membrane potential and led to a drastic increase in green fiuorescence in two strains (Figures 4A,B). Further experiments showed that CT caused a dose-dependent and rapid dissipation of bacterial membrane potential of within 1 min of the addition of this agent (Figures 4C,D). CT rapidly dissipated bacterial membrane potential without causing severe membrane damage, suggesting that this compound is a respiratory chain inhibitor.

### CT Appears to Act as a Respiratory Chain Inhibitor Probably by Targeting NDH-2

**FIGURE 5:**
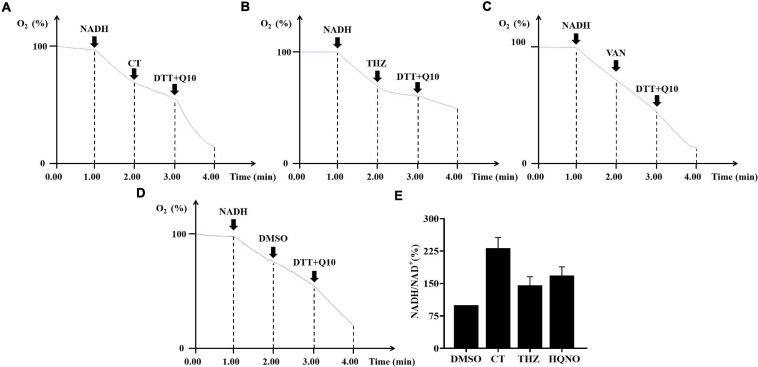
The effects of CT on respiratory chain activity of bacteria. **(A–D)** Inhibition of oxygen consumption of staphylococcal membrane vesicles by CT. Respiration was initiated by the addition of 1 mM NADH. Thioridazine (THZ) was used as positive control. Vancomycin (VAN) and DMSO were used as negative control, respectively. CT, 80 μg/mL; THZ, 100 μg/mL; VAN, 64 μg/mL; DMSO, 2%; dithiothreitol (DTT), 2.85 mM; ubiquinone-10 (Q10), 40 μM. **(E)** Effect of CT on NADH/NAD^+^ ratios. Intracellular NADH/NAD^+^ ratios of *S. aureus* ATCC 43300 were determined by an Amplite fluorimetric NAD/NADH ratio assay kit. DMSO, 3.2%; CT, 32 μg/mL; thioridazine (THZ), 64 μg/mL; HQNO, 32 μg/mL.

The oxygen consumption of membrane vesicles derived from *S. aureus* was determined in a cell-free assay to test whether CT is a respiratory chain inhibitor. An immediate linear consumption of oxygen was observed when NADH was added to the staphylococcal membrane vesicles. The oxygen consumption was immediately inhibited by the addition of CT (Figure 5A), confirming that this agent is an inhibitor of respiratory systems. Similar results were observed with the addition of thioridazine (Figure 5B). As negative controls, vancomycin (Figure 5C) or DMSO (Figure 5D) did not inhibit the oxygen consumption initiated by NADH. Interestingly, respiration in the CT-arrested membranes was restored by the addition of dithiothreitol/ubiquinone-10. It was previously reported that there is no gene encoding type-1 NADH:quinone oxidoreductase, bc_1_ complex, and cytochrome *c* oxidase in the genome of *S. aureus*, and that NDH-2 is the sole NADH:quinone oxidoreductase expressed in this strain (Schurig-Briccio et al., 2014). Thus, it was reasonable to speculate that the primary site of inhibition of respiration by CT is NDH-2. In agreement with the hypothesis, CT prevented efficient reoxidization of NADH to NAD^+^, which resulted in a substantially increased NADH/NAD^+^ ratio in CT-treated *S. aureus* (Figure 5E). The NAD^+^ level in *B. subtilis* treated with CT also decreased significantly revealed by the metabonomic analysis described above.

**FIGURE 6:**
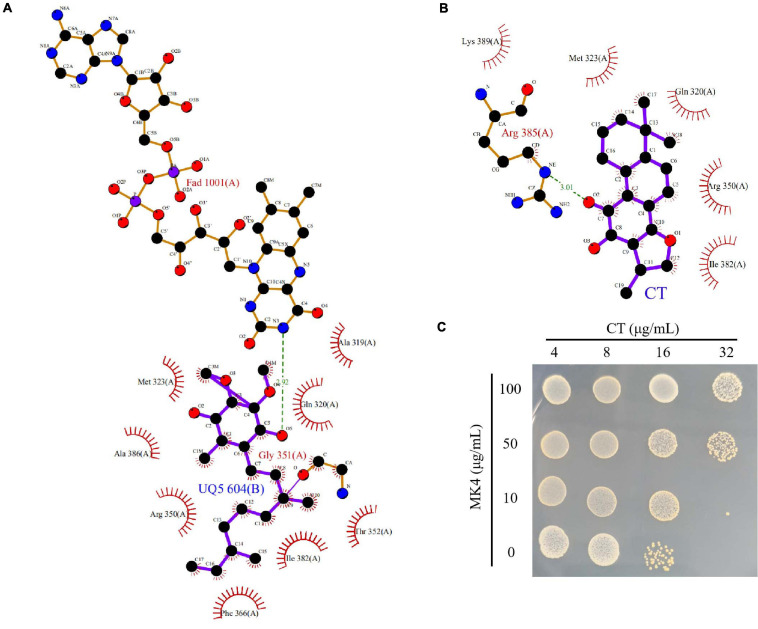
CT competitively inhibits the binding of quinone to NDH-2. **(A)** Configuration for the interaction of ubiquinone-5 (UQ5) with NDH-2. **(B)** Configuration for the interaction of CT with NDH-2. **(C)** MK4 antagonizes the antibacterial activity of CT.

To understand the interaction mode of CT binding to NDH-2, the molecular docking was performed. As shown in Figure 6A, ubiquinone interacted with Ala319, Gln320, Met323, Arg350, Thr352, Phe366, Ile382, and Ala386 *via* hydrophobic contact, and generated hydrogen bond interaction with fiavin adenine dinucleotide (FAD), which is another important cofactor for NDH-2 (Schurig-Briccio et al., 2014). This observation was consistent with the proposal that the quinone binding cavity is near the *si*-side of FAD, and lined mostly by hydrophobic residues, namely, Tyr15, Ala319, Met323, Ile382, and Ala386 (Heikal et al., 2014; Sena et al., 2015). Intriguingly, CT was found to form a hydrogen bond with Arg385 (Figure 6B), which is located at the tunnel entrance of the quinone binding cavity in *S. aureus* NDH-2 (Sena et al., 2015). Besides, NDH-2 interacted with CT *via* hydrophobic contact through residues Gln320, Met323, Arg350, Ile382, and Lys389, which are partially overlapped with the binding sites of quinone. Thus, we speculated that CT could competitively inhibit the binding of quinone to NDH-2. In agreement with this hypothesis, supplementing CT-treated cultures with MK4 rescued the CT-treated bacteria (Figure 6C and Table 2). Interestingly, an antagonistic effect was observed in the combination of MK4 with 2-n-heptyl-4-hydroxyquinoline-*N*-oxide (HQNO), but not thioridazine (Table 2), both of which were reported to be NDH-2 inhibitors with different mechanisms (Schurig-Briccio et al., 2014; Sena et al., 2015).

**TABLE 2:** Effects of menaquinone on antibacterial activity of CT.

**Indicator strain**	**MIC (μg/mL)**					
	**CT**	**CT + MK4^a^**	**HQNO**	**HQNO + MK4^a^**	**THZ**	**THZ + MK4^a^**
*S. aureus* ATCC 43300	8	>32	8	32	32	32
*B. subtilis* 168	4	>32	–	–	–	–

*^*a*^MK4 (menaquinone-4) concentration is 100 μg/mL.*

### CT Inhibits the Respiratory ATP Synthesis of Bacteria

**FIGURE 7:**
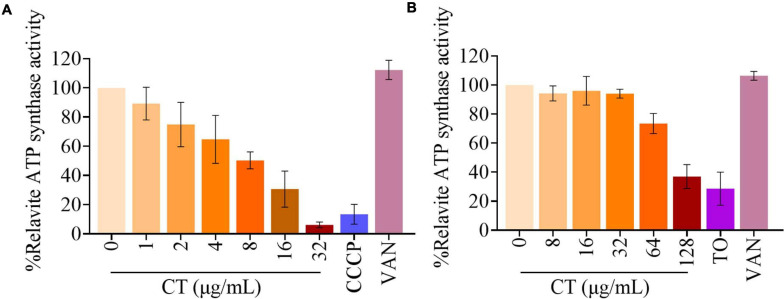
Effect of CT on ATP synthesis. **(A)** Cellular ATP levels in *B. subtilis* 168 were measured in the presence of CT at specified concentration after the addition of 10 mM glucose for 30 min. Vancomycin (VAN, 2 μg/mL) and CCCP (8 μg/mL) were used as negative and positive control, respectively. **(B)** The ATP synthesis activity was determined in membrane vesicles isolated from *S. aureus* ATCC 43300 energized with NADH. Vancomycin (VAN, 32 μg/mL) and Tomatidine (TO, 64 μg/mL) were used as negative and positive control, respectively. Data from three independent experiments are presented as mean ± SD.

Blocking the access of electrons into the respiratory chain achieved by inhibiting essential enzyme NDH-2 normally prevents the synthesis of ATP. Thus, we investigated the impact of inhibition of ATP synthesis by CT on cellular ATP levels. As shown in Figure 7A, a significant decrease in ATP levels was observed in CT-treated *B. subtilis* cells compared to the controls. The 50% inhibitory concentration (IC_50_) of CT against *B. subtilis* was found to be 9.6 ± 1.3 μg/mL. However, CT did not significantly change ATP levels in *S. aureus* ATCC 43300 (Supplementary Figure 3). To eliminate the interference from ATP synthesized by phosphorylation at the substrate level, we determined the inhibitory activities of CT on ATP synthesis in staphylococcal membrane vesicles. As depicted in Figure 7B, CT showed remarkable inhibitory activity with an IC_50_ value of 107.1 ± 8.6 μg/mL, indicating that it possessed the ability to inhibit the respiratory ATP synthesis of *S. aureus*.

### CT Interactions With Other Respiratory Chain Inhibitors

Synergistic interactions have been reported between molecules targeting different components of the bacterial respiratory chain (Berube and Parish, 2017; Foo et al., 2018; Iqbal et al., 2018; Lu et al., 2019). Thus, checkerboard assays for two-drug combinations were performed to determine the nature of interactions between CT with HQNO, thioridazine, 7-Methoxy-2-naphthol (MenA inhibitor) (Choi et al., 2016), or tomatidine (ATP Synthase inhibitor) (Lamontagne Boulet et al., 2018) in *S. aureus* ATCC 43300. As shown in Table 3, the interaction of CT with HQNO was additive with a fractional inhibitory concentration index (FICI) value of 0.75. The combinations of CT with 7-Methoxy-2-naphthol and thioridazine showed promising synergy with FICI values of 0.31 and 0.37, respectively. A strong synergy between CT and tomatidine with FICI of <0.19 was also observed.

**TABLE 3:** Interactions between CT and other respiratory chain inhibitors against *S. aureus* ATCC 43300.

**Drug combination**	**Alone**	**Combination**	**FICI**	**Category^a^**
CT	8	2	0.75	Additive
HQNO	8	4		
CT	8	1	0.37	Synergistic
Thioridazine	32	8		
CT	8	0.5	0.31	Synergistic
7-Methoxy-2-naphthol	512	128		
CT	8	0.25	<0.19	Synergistic
Tomatidine	>64	10		

*^*a*^FICI categories: ≤0.5, synergistic; >0.5 to ≤1, additive; >1 to < 4, no interaction; ≥4, antagonism.*

## Discussion

Plant-derived antimicrobial agents have received much attention due to their effectiveness against drug-resistant strains. Thousands of compounds have been listed as antimicrobial phytochemicals (Ríos and Recio, 2005; Radulovic et al., 2013; Barbieri et al., 2017). However, most of the MICs of these plant metabolites are much higher than antibiotics commercially available, restricting their clinical application. One promising approach to improve the antimicrobial activity of phytochemicals involves the combination of different bioactive compounds. A deep understanding of the mechanism of drug action is conducive to the rational combination of antimicrobial agents. In this study, the antibacterial mechanism of CT and its effect of combination therapy were investigated.

Despite rapid technological progress, the identification of mechanisms of drug action is still a difficult task (Nonejuie et al., 2013; Zampieri, 2018; Martin et al., 2020). Metabolomics can detect dynamic changes in the abundance of thousands of small molecules in response to drug treatment and provide a deep insight into the mechanism of drug action (Zampieri, 2018). To obtain a first hint regarding the mechanism of action of CT, an untargeted metabolomics study of *B. subtilis* was conducted. The CT treatment induced significant changes in a wide range of glycerophospholipids. Similar alterations of the major membrane phospholipids were observed when bacteria were treated with surfactin or polymyxin B (Uttlová et al., 2016; Tran et al., 2018), both of which are membrane-damaging antimicrobials. Thus, we speculated that CT is also an antibacterial agent targeting the cell membrane. Unexpectedly, CT was subsequently proved to be a compound targeting the function of the membrane-associated respiratory chain rather than the organization of the bacterial membrane bilayer.

The most striking feature of *B. subtilis* cells treated with CT was the reduced level of PG accompanied by an increase in the content of PE and PA. These phospholipids vary not only in the length of fatty acids but also in the composition of their headgroups, which differ significantly in charge and propensity to form non-bilayer structures (Salzberg and Helmann, 2008). PA has been shown to stabilize and bind stronger than PG *via* H-bonds/electrostatic interactions with the membrane proteins, such as KcsA (Raja et al., 2007). PE was also reported to facilitate membrane association and insertion of monomeric KcsA, due to its small headgroup and extensive hydrogen bonding properties (Raja, 2011). Thus, we suggest that the increased levels of PA and PE contribute to the assembly and stabilization of membrane-bound respiratory chain proteins, which may counteract the destabilizing effect induced by CT. The altered compositions of phospholipid may also impede entry of CT into the membrane, hindering access to its target. The relationship between the altered compositions of membrane phospholipid and the inhibition of the respiratory chain activity in bacteria treated with CT will need further study.

Cryptotanshinone rapidly dissipated bacterial membrane potential without causing severe membrane damage, suggesting that this compound is a respiratory chain inhibitor. It is documented that NADH produced in the cytoplasm of *S. aureus* is reoxidized to NAD^+^ mainly by NDH-2, the sole type of NADH:quinone oxidoreductase expressed in this strain (Schurig-Briccio et al., 2014; Sena et al., 2015). Menaquinol generated from the corresponding menaquinone by NDH-2 is then directly oxidized by terminal oxidases (Schurig-Briccio et al., 2014). Taking advantage of the simple organization of the staphylococcal respiratory chain, we used the membrane vesicles derived from *S. aureus* to investigate the effect of CT on respiratory activity by oxygen consumption assay. The NADH-driven oxygen consumption was immediately inhibited by the addition of CT, confirming that this agent is an inhibitor of respiratory systems. Interestingly, respiration in the CT-arrested membranes was restored by the addition of dithiothreitol/ubiquinone-10, which donated electrons at the level of quinol:oxygen oxidoreductase. The recovery of oxygen consumption implied that the site of inhibition by CT is NDH-2, which is the only NADH dehydrogenase upstream of terminal quinol:oxygen oxidoreductase in *S. aureus* (Schurig-Briccio et al., 2014). This is supported by the observation that the NADH/NAD^+^ ratio was substantially increased in CT-treated *S. aureus*.

Type II NADH dehydrogenase is a membrane bound protein with a non-covalently bound FAD as a cofactor, which catalyses the cytoplasmic oxidation of NADH and reduction of quinone in the membrane. The crystal structure of NDH-2 from *S. aureus* suggested that NADH and the quinone bind to different sites of the enzyme (Sena et al., 2015). The docking analysis showed that the binding sites of CT to NDH-2 partially overlap with that of quinone, implying that CT would competitively inhibit the binding of quinone to NDH-2. Consistent with this speculation, the antimicrobial activity of CT against *B. subtilis* and *S. aureus* was blocked by MK4. Some antimicrobial agents mainly targeting NDH-2 have been extensively described. Among them, HQNO was found to competitively inhibit binding of a quinone substrate for *S*. *aureus* NDH-2 (Sena et al., 2015; Petri et al., 2018), while phenothiazine compounds were reported to act as non-competitive inhibitors for quinone (Yano et al., 2006). In agreement with the literature, MK4 supplementation blocked the anti-*S. aureus* activity of HQNO but not phenothiazine thioridazine (Table 2). The observations that the combination of CT with thioridazine but not HQNO, exerted synergistic activity against *S. aureus* (Table 3), further implied that CT might act as a competitive inhibitor with respect to quinone.

Type II NADH dehydrogenase is an important enzyme in the respiratory system of many organisms, including *S. aureus* and *B. subtilis*. It is a primary entry point for electrons into the electron transport chain for generation of ATP, and is responsible for maintaining cellular NAD^+^/NADH balance. Blocking the introduction of electrons into the respiratory chain achieved by inhibition of the enzyme NDH-2 can indirectly dissipate proton motive force, which is composed of the membrane potential and the transmembrane proton gradient. The proton motive force is essentially involved in the process of respiratory ATP synthesis and the active transport of solutes such as amino acids (Allen et al., 1991; Hards and Cook, 2018). This could be the reason why CT not only disrupted the NAD^+^/NADH balance and proton motive force but also inhibited respiratory ATP synthesis.

## Conclusion

In general, the direct and indirect evidence described above supports that CT is a respiratory chain inhibitor probably by targeting NDH-2, although more research is needed to confirm this. CT rapidly dissipates bacterial membrane potential and disrupts the NAD^+^/NADH balance without causing significant membrane damage. Menaquinone antagonizes antibacterial activity of CT, while synergistic activities can be achieved by the combinations of CT with thioridazine, 7-Methoxy-2-naphthol, or tomatidine, but not HQNO. All these observations are consistent with the finding that CT is a respiratory chain inhibitor. However, the mechanisms of its antibacterial activity should be investigated further.

## Data Availability Statement

The original contributions presented in the study are included in the article/Supplementary Material. Further inquiries can be directed to the corresponding author/s.

## Author Contributions

C-DQ, B-CC, and Z-SD designed the experiments. B-CC, J-SD, N-PC, X-WG, and L-FM conducted the experiments. C-DQ, B-CC, Z-SD, J-SD, N-PC, X-WG, and L-FM analyzed the data. C-DQ, B-CC, and Z-SD wrote the manuscript. All authors read and approved the final version of the manuscript.

## Conflict of Interest

The authors declare that the research was conducted in the absence of any commercial or financial relationships that could be construed as a potential conflict of interest.

## Footnotes

1 http://www.rcsb.org/

## Supplementary Material

Supplementary Figure 1 Score scatter plots of PCA, PLS-DA, OPLS-DA, and validation plots of OPLS-DA for two comparative groups. **(A)** PCA for CT/Con comparative group. **(B)** PLS-DA for CT/Con comparative group. **(C)** OPLS-DA for CT/Con comparative group. **(D)** Validation plots of OPLS-DA for CT/Con comparative group. CT, 16 μg/mL cryptotanshinone-treated group; Con, 1.6% DMSO-treated group.

Supplementary Figure 2 Histogram of the different pathway −log (*P* = 10) values enriched in the CT-treated strain according to KEGG pathway analysis. The horizontal line (blue) at 1.3 indicates *P* < 0.05; the horizontal line (red) at 2 indicates *P* < 0.01.

Supplementary Figure 3 Effect of CT on ATP synthesis. Cellular ATP levels in *S. aureus* ATCC 43300 were measured in the presence of CT at specified concentration after the addition of 10 mM glucose for 30 min. Vancomycin (VAN, 8 μg/mL) and CCCP (8 μg/mL) were used as negative and positive control, respectively.

Supplementary Data Set 1 Y1-6 is cryptotanshinone-treated group.

Supplementary Data Set 2 D1-6 is DMSO-treated group.

## References

[B1] Allen N. E. Alborn W. E. Hobbs J. N. (1991). Inhibition of membrane potential-dependent amino acid transport by daptomycin. *Antimicrob. Agents Chemother.* 35 2639–2642. 10.1128/AAC.35.12.2639 1687346 PMC245446

[B2] Al-Mebairik N. F. El-Kersh T. A. Al-Sheikh Y. A. Marie M. A. M. (2016). A review of virulence factors, pathogenesis, and antibiotic resistance in Staphylococcus aureus. *Rev. Med. Microbiol.* 27 50–56. 10.1097/MRM.0000000000000067

[B3] Andries K. (2005). A Diarylquinoline Drug Active on the ATP Synthase of Mycobacterium tuberculosis. *Science* 307 223–227. 10.1126/science.1106753 15591164

[B4] Anraku Y. (1988). Bacterial Electron Transport Chains. *Annu. Rev. Biochem.* 57 101–132. 10.1146/annurev.bi.57.070188.000533 3052268

[B5] Azarkina N. Siletsky S. Borisov V. von Wachenfeldt C. Hederstedt L. Konstantinov A. A. (1999). A Cytochrome bb ‘-type Quinol Oxidase in Bacillus subtilis Strain 168. *J. Biol. Chem.* 274 32810–32817. 10.1074/jbc.274.46.32810 10551842

[B6] Barbieri R. Coppo E. Marchese A. Daglia M. Sobarzo-Sánchez E. Nabavi S. F. (2017). Phytochemicals for human disease: An update on plant-derived compounds antibacterial activity. *Microbiol. Res.* 196 44–68. 10.1016/j.micres.2016.12.003 28164790

[B7] Berube B. J. Parish T. (2017). Combinations of Respiratory Chain Inhibitors Have Enhanced Bactericidal Activity against Mycobacterium tuberculosis. *Antimicrob. Agents Chemother.* 62:1677. 10.1128/AAC.01677-17 29061760 PMC5740367

[B8] Boersch M. Rudrawar S. Grant G. Zunk M. (2018). Menaquinone biosynthesis inhibition: a review of advancements toward a new antibiotic mechanism. *RSC Adv.* 8 5099–5105. 10.1039/C7RA12950E PMC9078190 35542397

[B9] Cha J.-D. Lee J.-H. Choi K. M. Choi S.-M. Park J. H. (2014). Synergistic Effect between Cryptotanshinone and Antibiotics against Clinic Methicillin and Vancomycin-Resistant Staphylococcus aureus. *Evid. Based Compl. Altern. Med.* 2014 1–16. 10.1155/2014/450572 24782909 PMC3982256

[B10] Chen B.-C. Lin C.-X. Chen N.-P. Gao C.-X. Zhao Y.-J. Qian C.-D. (2018). Phenanthrene Antibiotic Targets Bacterial Membranes and Kills Staphylococcus aureus With a Low Propensity for Resistance Development. *Front. Microbiol.* 9:1593. 10.3389/fmicb.2018.01593 30065715 PMC6056686

[B11] Choi S. Larson M. A. Hinrichs S. H. Bartling A. M. Frandsen J. Narayanasamy P. (2016). Discovery of bicyclic inhibitors against menaquinone biosynthesis. *Future Med. Chem.* 8 11–16. 10.4155/fmc.15.168 26699277 PMC5558544

[B12] Clinical and Laboratory Standards Institute (2009). *Methods for dilution antimicrobial susceptibility tests for bacteria that grow aerobically. Approv. Stand. (8th ed.).* Wayne, PA: CLSI, publication.

[B13] Feng H. Xiang H. Zhang J. Liu G. Guo N. Wang X. (2009). Genome-Wide Transcriptional Profiling of the Response of Staphylococcus aureus to Cryptotanshinone. *J. Biomed. Biotechnol.* 2009 1–8. 10.1155/2009/617509 19707532 PMC2730559

[B14] Foo C. S. Lupien A. Kienle M. Vocat A. Benjak A. Sommer R. (2018). Arylvinylpiperazine Amides, a New Class of Potent Inhibitors Targeting QcrB of Mycobacterium tuberculosis. *MBio* 9:18. 10.1128/mBio.01276-18 30301850 PMC6178619

[B15] Hamamoto H. Urai M. Ishii K. Yasukawa J. Paudel A. Murai M. (2015). Lysocin E is a new antibiotic that targets menaquinone in the bacterial membrane. *Nat. Chem. Biol.* 11 127–133. 10.1038/nchembio.1710 25485686

[B16] Hards K. Cook G. M. (2018). Targeting bacterial energetics to produce new antimicrobials. *Drug Resist. Updat.* 36 1–12. 10.1016/j.drup.2017.11.001 29499834

[B17] Heikal A. Nakatani Y. Dunn E. Weimar M. R. Day C. L. Baker E. N. (2014). Structure of the bacterial type II NADH dehydrogenase: a monotopic membrane protein with an essential role in energy generation. *Mol. Microbiol.* 91 950–964. 10.1111/mmi.12507 24444429

[B18] Hurdle J. G. O’Neill A. J. Chopra I. Lee R. E. (2011). Targeting bacterial membrane function: an underexploited mechanism for treating persistent infections. *Nat. Rev. Microbiol.* 9 62–75. 10.1038/nrmicro2474 21164535 PMC3496266

[B19] Iqbal I. Bajeli S. Akela A. Kumar A. (2018). Bioenergetics of Mycobacterium: An Emerging Landscape for Drug Discovery. *Pathogens* 7:24. 10.3390/pathogens7010024 29473841 PMC5874750

[B20] Koul A. Dendouga N. Vergauwen K. Molenberghs B. Vranckx L. Willebrords R. (2007). Diarylquinolines target subunit c of mycobacterial ATP synthase. *Nat. Chem. Biol.* 3 323–324. 10.1038/nchembio884 17496888

[B21] Lamontagne Boulet M. Isabelle C. Guay I. Brouillette E. Langlois J.-P. Jacques P. -É, et al. (2018). Tomatidine Is a Lead Antibiotic Molecule That Targets Staphylococcus aureus ATP Synthase Subunit C. *Antimicrob. Agents Chemother.* 62:2197. 10.1128/AAC.02197-17 29610201 PMC5971568

[B22] Lee D.-S. Lee S.-H. Noh J.-G. Hong S.-D. (1999). Antibacterial Activities of Cryptotanshinone and Dihydrotanshinone I from a Medicinal Herb, Salvia miltiorrhiza Bunge. *Biosci. Biotechnol. Biochem.* 63 2236–2239. 10.1271/bbb.63.2236 10664860

[B23] Lu G. (2020). The Mechanism in the Protection of Cryptotanshinone against Infection by Listeria monocytogenes.

[B24] Lu X. Williams Z. Hards K. Tang J. Cheung C.-Y. Aung H. L. (2019). Pyrazolo[1,5- a]pyridine Inhibitor of the Respiratory Cytochrome bcc Complex for the Treatment of Drug-Resistant Tuberculosis. *ACS Infect. Dis.* 5 239–249. 10.1021/acsinfecdis.8b00225 30485737

[B25] Martin J. K. Sheehan J. P. Bratton B. P. Moore G. M. Mateus A. Li S. H.-J. (2020). A Dual-Mechanism Antibiotic Kills Gram-Negative Bacteria and Avoids Drug Resistance. *Cell* 181 1518–1532.e14. 10.1016/j.cell.2020.05.005 32497502 PMC7780349

[B26] Melo A. M. P. Bandeiras T. M. Teixeira M. (2004). New Insights into Type II NAD(P)H:Quinone Oxidoreductases. *Microbiol. Mol. Biol. Rev.* 68 603–616. 10.1128/MMBR.68.4.603-616.2004 15590775 PMC539002

[B27] Müller A. Wenzel M. Strahl H. Grein F. Saaki T. N. V. Kohl B. (2016). Daptomycin inhibits cell envelope synthesis by interfering with fluid membrane microdomains. *Proc. Natl. Acad. Sci.* 113 E7077–E7086. 10.1073/pnas.1611173113 27791134 PMC5111643

[B28] Nonejuie P. Burkart M. Pogliano K. Pogliano J. (2013). Bacterial cytological profiling rapidly identifies the cellular pathways targeted by antibacterial molecules. *Proc. Natl. Acad. Sci.* 110 16169–16174. 10.1073/pnas.1311066110 24046367 PMC3791758

[B29] Novo D. J. Perlmutter N. G. Hunt R. H. Shapiro H. M. (2000). Multiparameter Flow Cytometric Analysis of Antibiotic Effects on Membrane Potential, Membrane Permeability, and Bacterial Counts of Staphylococcus aureus andMicrococcus luteus. *Antimicrob. Agents Chemother.* 44 827–834. 10.1128/AAC.44.4.827-834.2000 10722477 PMC89778

[B30] Paudel A. Hamamoto H. Panthee S. Sekimizu K. (2016). Menaquinone as a potential target of antibacterial agents. *Drug Discov. Ther.* 10 123–128. 10.5582/ddt.2016.01041 27431268

[B31] Petri J. Shimaki Y. Jiao W. Bridges H. R. Russell E. R. Parker E. J. (2018). Structure of the NDH-2 – HQNO inhibited complex provides molecular insight into quinone-binding site inhibitors. *Biochim. Biophys. Acta Bioenerg.* 1859 482–490. 10.1016/j.bbabio.2018.03.014 29621505 PMC6167311

[B32] Radulovic N. S. Blagojevic P. D. Stojanovic-Radic Z. Z. Stojanovic N. M. (2013). Antimicrobial Plant Metabolites: Structural Diversity and Mechanism of Action. *Curr. Med. Chem.* 20 932–952. 10.2174/092986713805219136 23210781

[B33] Raja M. (2011). Do Small Headgroups of Phosphatidylethanolamine and Phosphatidic Acid Lead to a Similar Folding Pattern of the K+ Channel? *J. Membr. Biol.* 242 137–143. 10.1007/s00232-011-9384-4 21744243 PMC3146712

[B34] Raja M. Spelbrink R. E. J. de Kruijff B. Killian J. A. (2007). Phosphatidic acid plays a special role in stabilizing and folding of the tetrameric potassium channel KcsA. *FEBS Lett.* 581 5715–5722. 10.1016/j.febslet.2007.11.039 18036565

[B35] Ríos J. L. Recio M. C. (2005). Medicinal plants and antimicrobial activity. *J. Ethnopharmacol.* 100 80–84. 10.1016/j.jep.2005.04.025 15964727

[B36] Saising J. Nguyen M.-T. Härtner T. Ebner P. Al Mamun, Bhuyan A. (2018). Rhodomyrtone (Rom) is a membrane-active compound. *Biochim. Biophys. Acta Biomembr.* 1860 1114–1124. 10.1016/j.bbamem.2018.01.011 29317198

[B37] Salzberg L. I. Helmann J. D. (2008). Phenotypic and Transcriptomic Characterization of Bacillus subtilis Mutants with Grossly Altered Membrane Composition. *J. Bacteriol.* 190 7797–7807. 10.1128/JB.00720-08 18820022 PMC2583605

[B38] Sarathy J. P. Ragunathan P. Cooper C. B. Upton A. M. Grüber G. Dick T. (2019). TBAJ-876 Displays Bedaquiline-Like Mycobactericidal Potency without Retaining the Parental Drug’s Uncoupler Activity. *Antimicrob. Agents Chemother.* 64:1540. 10.1128/AAC.01540-19 31712198 PMC6985740

[B39] Schurig-Briccio L. A. Yano T. Rubin H. Gennis R. B. (2014). Characterization of the type 2 NADH:menaquinone oxidoreductases from Staphylococcus aureus and the bactericidal action of phenothiazines. *Biochim. Biophys. Acta Bioenerg.* 1837 954–963. 10.1016/j.bbabio.2014.03.017 24709059 PMC4047164

[B40] Sena F. V. Batista A. P. Catarino T. Brito J. A. Archer M. Viertler M. (2015). Type-II NADH:quinone oxidoreductase from S taphylococcus aureus has two distinct binding sites and is rate limited by quinone reduction. *Mol. Microbiol.* 98 272–288. 10.1111/mmi.13120 26172206

[B41] Teng Z. Li M. Shi D. Deng X. Wang J. (2018). Synergistic interactions of cryptotanshinone and aminoglycoside antibiotics against Staphylococcus aureus in vitro. *J. Glob. Antimicrob. Resist.* 13 264–265. 10.1016/j.jgar.2018.05.013 29807203

[B42] Tran T. B. Bergen P. J. Creek D. J. Velkov T. Li J. (2018). Synergistic Killing of Polymyxin B in Combination With the Antineoplastic Drug Mitotane Against Polymyxin-Susceptible and -Resistant *Acinetobacter baumannii*: A Metabolomic Study. *Front. Pharmacol.* 9:359. 10.3389/fphar.2018.00359 29713282 PMC5911485

[B43] Uttlová P. Pinkas D. Bechyòková O. Fišer R. Svobodová J. Seydlová G. (2016). Bacillus subtilis alters the proportion of major membrane phospholipids in response to surfactin exposure. *Biochim. Biophys. Acta Biomembr.* 1858 2965–2971. 10.1016/j.bbamem.2016.09.006 27620333

[B44] Weinstein E. A. Yano T. Li L.-S. Avarbock D. Avarbock A. Helm D. (2005). Inhibitors of type II NADH:menaquinone oxidoreductase represent a class of antitubercular drugs. *Proc. Natl. Acad. Sci.* 102 4548–4553. 10.1073/pnas.0500469102 15767566 PMC555520

[B45] Yano T. Li L.-S. Weinstein E. Teh J.-S. Rubin H. (2006). Steady-state Kinetics and Inhibitory Action of Antitubercular Phenothiazines on Mycobacterium tuberculosis Type-II NADH-Menaquinone Oxidoreductase (NDH-2). *J. Biol. Chem.* 281 11456–11463. 10.1074/jbc.M508844200 16469750

[B46] Zampieri M. (2018). From the metabolic profiling of drug response to drug mode of action. *Curr. Opin. Syst. Biol.* 10 26–33. 10.1016/j.coisb.2018.05.005

[B47] Zhang G. Wu Y. Zhang H. Gong X. (2003). Comparative evaluation of Kecuo Yintong Cream with 1% clindamycin gel in the treatment of acne vulgaris. *J. Clin. Dermatol.* 32 356–357.

[B48] Zhao H. Zhang H. Shen Y. (2007). Application of tanshinone capsules in dermatosis. *Med. Recapitul.* 13 2048–2049.

