# Supplementary figures and images for: New Insights Into the Antibacterial Mechanism of Cryptotanshinone, a Representative Diterpenoid Quinone From Salvia miltiorrhiza Bunge
Source: Front Microbiol. 2021 Feb 25;12:647289. doi: 10.3389/fmicb.2021.647289 (PMC7950322; doi:10.3389/fmicb.2021.647289)

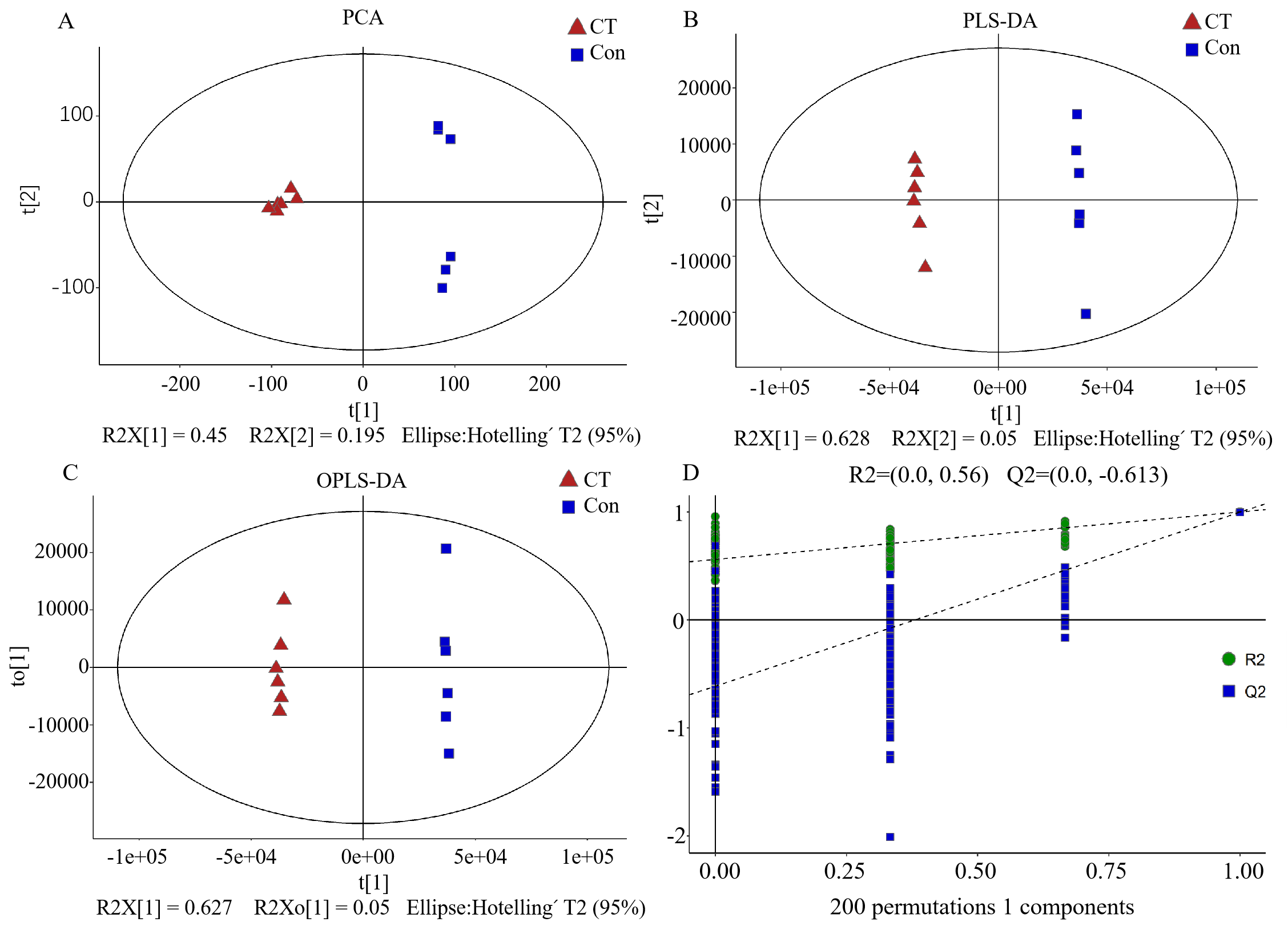

Supplement: Supplementary Figure 1 — Score scatter plots of PCA, PLS-DA, OPLS-DA, and validation plots of OPLS-DA for two comparative groups. (A) PCA for CT/Con comparative group. (B) PLS-DA for CT/Con comparative group. (C) OPLS-DA for CT/Con comparative group. (D) Validation plots of OPLS-DA for CT/Con comparative group. CT, 16 μg/mL cryptotanshinone-treated group; Con, 1.6% DMSO-treated group. [file Image_1.tiff]

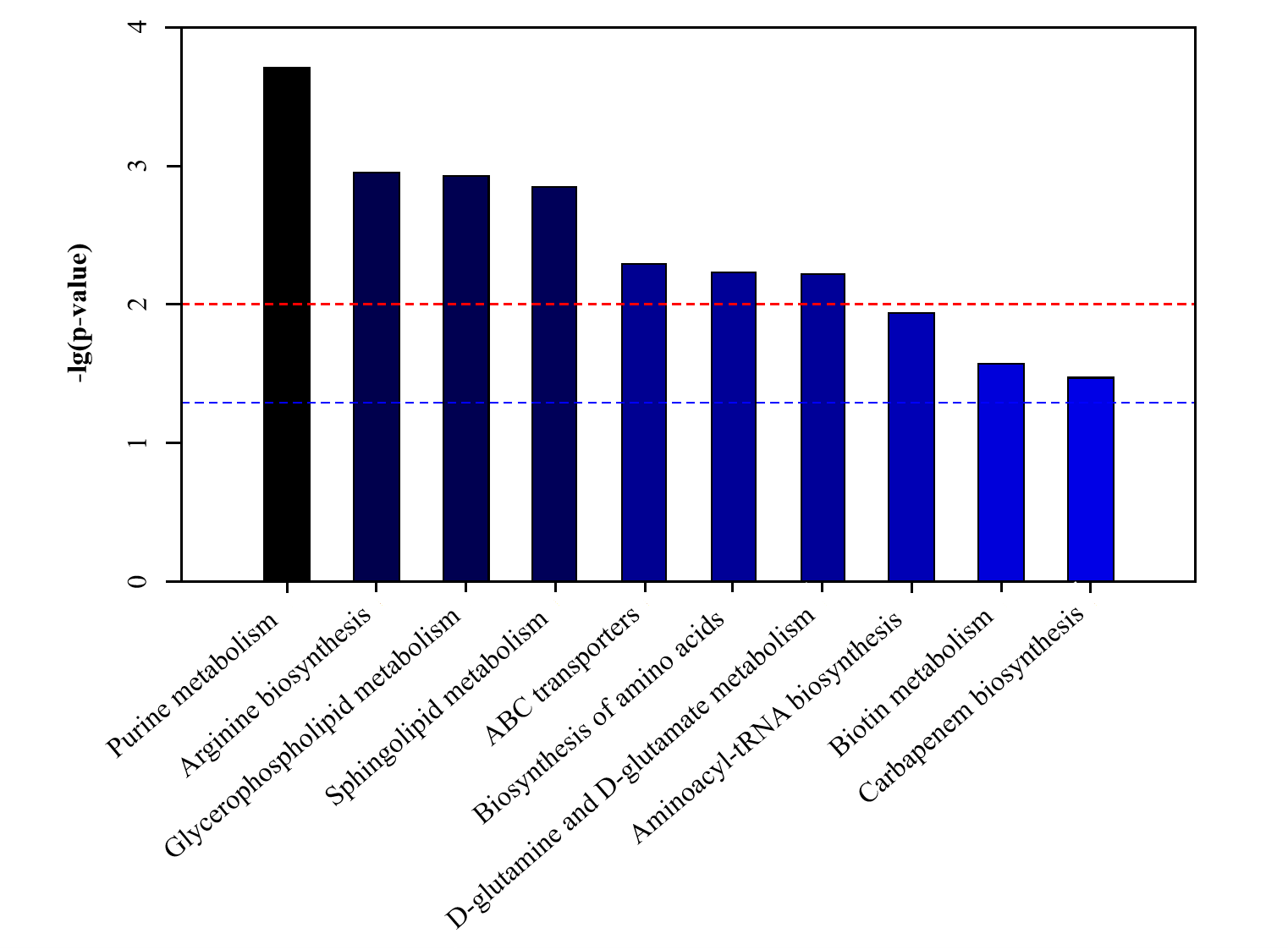

Supplement: Supplementary Figure 2 — Histogram of the different pathway −log (P = 10) values enriched in the CT-treated strain according to KEGG pathway analysis. The horizontal line (blue) at 1.3 indicates P < 0.05; the horizontal line (red) at 2 indicates P < 0.01. [file Image_2.tiff]

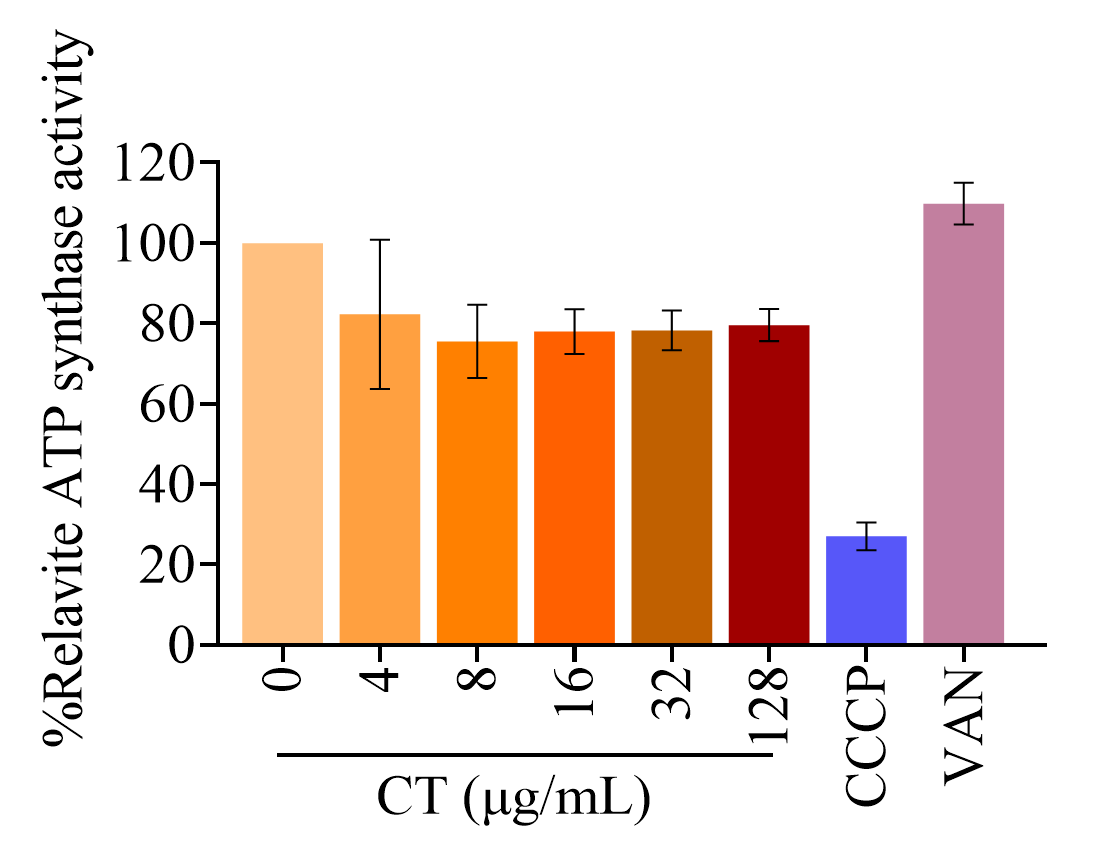

Supplement: Supplementary Figure 3 — Effect of CT on ATP synthesis. Cellular ATP levels in S. aureus ATCC 43300 were measured in the presence of CT at specified concentration after the addition of 10 mM glucose for 30 min. Vancomycin (VAN, 8 μg/mL) and CCCP (8 μg/mL) were used as negative and positive control, respectively. [file Image_3.tif]
